# A Pediatric Case of Ramsay Hunt Syndrome

**DOI:** 10.1155/2014/469565

**Published:** 2014-09-08

**Authors:** Serhan Derin, Hatice Derin, Murat Sahan, Hüseyin Çaksen

**Affiliations:** ^1^Muğla Sıtkı Koçman Üniversitesi, Tıp Fakültesi, Orhaniye Mahallesi, Haluk Özsoy Caddesi, 48000 Mugla, Turkey; ^2^Konya Necmettin Erbakan Üniversitesi, Meram Tıp Fakültesi, Yunus Emre Mahallesi, 42030 Konya, Turkey

## Abstract

Ramsay Hunt syndrome (RHS) is characterized by facial paralysis, inner ear dysfunction, periauricular pain, and herpetiform vesicles. The reported incidence in children is 2.7/100,000. The pathogenesis involves the reactivation of latent varicella zoster virus (VZV) in the geniculate ganglion of the facial nerve. The recovery rate is better in children than in adults. This paper discusses a 12-year-old girl with a rare case of peripheral facial paralysis caused by RHS and reviews the literature.

## 1. Introduction

In 1907, Hunt first described Ramsay Hunt syndrome (RHS), a disease characterized by facial paralysis, inner ear dysfunction, periauricular pain, and herpetiform vesicles [1]. Its etiology involves the reactivation of latent varicella zoster virus (VZV) in the geniculate ganglion cells [2]. Only 10% of RHS-associated facial paralysis cases are seen in children, and the reported incidence of RHS in patients younger than 10 years of age is 2.7/100.000 [3, 4].

Congenital anomalies, trauma, otitis media, Lyme disease, metabolic and genetic factors, and malignancy of the temporal bone or parotid gland can cause peripheral facial paralysis in children. Herpes simplex virus (HSV-1), Epstein-Barr virus (EBV), cytomegalovirus (CMV), human herpes virus (HHV 6 and 7), and mumps virus can all cause acute peripheral paralysis. However, the etiology is unknown in most cases, resulting in a diagnosis of idiopathic peripheral facial paralysis or Bell's palsy. Bell's palsy is the most common peripheral facial paralysis in children, and it is reported to cause 24–70% of all cases of peripheral facial paralysis in children [5].

Although RHS is the second most common atraumatic cause of facial paralysis, it is very rare in children. Hato et al. reported that RHS caused 16.7% of all peripheral facial palsies in children [6].

This study presents a case of peripheral facial paralysis caused by RHS and discusses the current literature.

## 2. Case Report

The patient was a 33 kg, 138 cm-tall, 12-year-old girl. She came to the pediatrics clinic with a chief complaint of facial asymmetry. She first developed a sore throat, cough, and runny nose, and this was followed 2 days later by pain around the right ear and a vesicular rash on the right ear pinna and behind the ear. The facial asymmetry started 3 days after the periauricular rash. She presented to our clinic on the second day of facial paralysis. She had no audiovestibular complaints, such as vertigo, tinnitus, nausea, vomiting, or hearing loss. Her medical history included varicella infection at 5 years of age.

**Figure 1:**
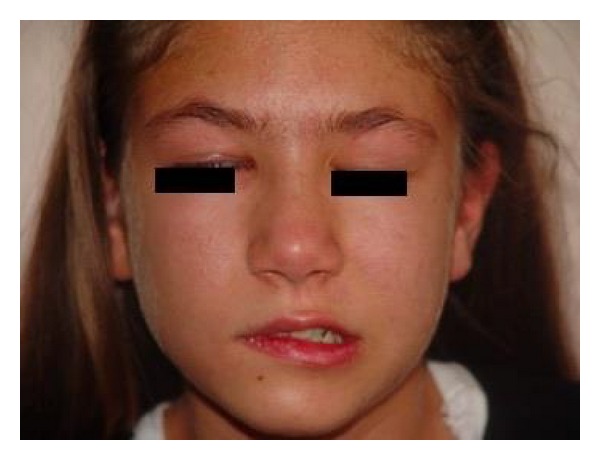
Grade 4 peripheral facial paralysis on the right.

**Figure 2:**
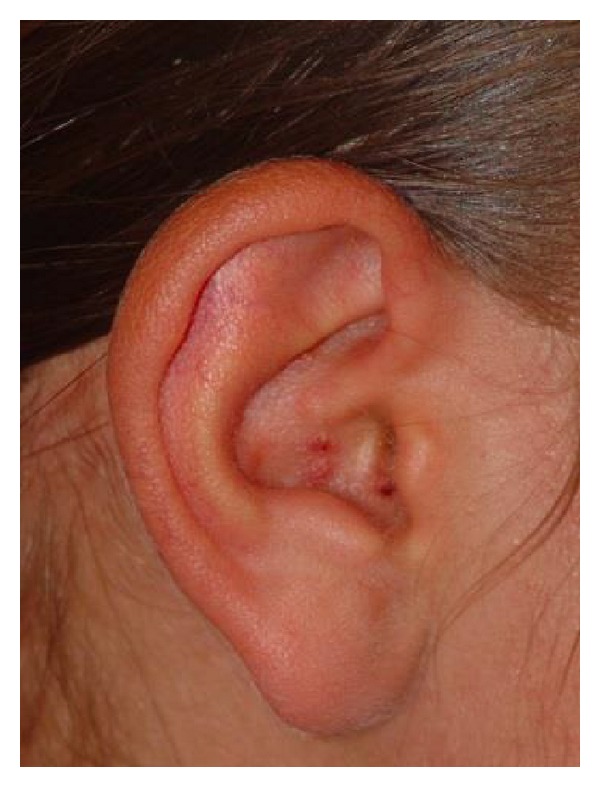
Crusted herpetic vesicles on the right ear pinna.

The patient had Grade 4 peripheral facial paralysis according to the House-Brackmann (HB) classification (Figure 1). She had crusting vesicular lesions on the right ear pinna and in the mastoid area (Figure 2). The physical examination showed crusted lesions in the outer ear canal; the tympanic membrane was normal. Results of the brainstem auditory evoked potential (BAEP) performed to evaluate eighth cranial nerve function were normal.

Based on the clinical findings, RHS was diagnosed at this point. The patient was started on methylprednisolone (2 mg/kg/day, maximum 60 mg/day) and oral acyclovir (800 mg/day) simultaneously. The methylprednisolone dose was tapered to 1 mg/kg at the beginning of the second week, tapered during the second week, and stopped on day 14. The acyclovir was continued for 5 days. Artificial tear drops were recommended to protect the cornea. Facial electromyography (EMG) was not required, as the patient's clinical picture improved by day 21. Cranial imaging was also not required, as the patient had no history of peripheral facial paralysis and no neurological manifestations.

## 3. Discussion

Herpes zoster oticus or Ramsay Hunt syndrome is a disease that affects cranial nerves 7 and 8. The acute peripheral facial paralysis is characterized by vestibulocochlear dysfunction and a herpetic rash around the ear pinna and outer ear canal. It is reported to be the cause of 2–10% of acute peripheral facial paralysis cases [6]. The reported incidence of VZV reactivation is higher in children 6–15 years of age than in younger children [7].

The primary VZV infection is varicella. The virus moves retrogradely and stays latent in the geniculate ganglion of the facial nerve, and the clinical picture of RHS presents after the reactivation of VZV there.

Typically, the clinical picture begins with otalgia that lasts 1~3 days. Then, herpetic vesicles appear in the outer ear canal, tympanic membrane, mouth, and distal 2/3 of the tongue, which can also cause losing taste sensation. If the eighth cranial nerve is also affected, nausea, vomiting, vertigo, nystagmus, tinnitus, and hearing loss can occur [8]. Hearing loss is reported in 24.4% of affected children [8]. Our patient had pain around the ear and mastoid bone 2 days before the vesicles appeared on the pinna. She had no vestibulocochlear nerve involvement, and the BAEP was normal.

The disease can also present with only acute peripheral facial paralysis, without the vesicular rash. This condition, termed “zoster sine herpete,” tends to be misdiagnosed as Bell's palsy [9].

To diagnose RHS in children, enzyme-linked immunosorbent assay (ELISA) serum anti-VZV IgG and IgM antibody titers are recommended [7]. In our case, these were not done because of the pathognomonic physical findings.

Starting steroids and antiviral agents in the early phase of the disease is recommended. Antivirals prevent VZV replication and facial nerve involvement, and the steroids prevent inflammation and edema. Murakami et al. examined the recovery of facial nerve function after starting treatment in the first 3 days, at 3–7 days, or later than 7 days and found that the recovery was best when treatment was started within 3 days of disease presentation [10]. We started the treatment on the second day of presentation, which was important for the clinical success. Early treatment is also important because complete facial nerve degeneration decreases the probability that facial nerve function will be recovered.

Kim and Bhimani recommended starting oral acyclovir at a dose of 800 mg for 7–10 days and prednisone 1 mg/kg/day for 5 days, followed by weaning over the next 6-7 days [11]. Kinishi et al. reported that the combination of acyclovir and steroid treatment was superior to treatment with acyclovir alone [12]. In our case, methylprednisolone and acyclovir were started simultaneously, and a marked improvement in facial nerve function was noted by day 21 (HB Grade II).

The prognosis of the facial paralysis in RHS is worse than that in Bell's palsy, and only 10% of complete facial paralysis in RHS recovers completely [13]. Furuta et al. reported a lower rate of recovery in RHS versus Bell's palsy (73% versus 100%) [7]. The prognosis for RHS in children is better than that in adults [6]. Audiovestibular findings, presentation including advanced facial paralysis, and starting treatment late result in a bad prognosis [5].

With this case report, we show that VZV reactivation can be a rare cause of acute peripheral facial paralysis in children aged 6–15 years and that early diagnosis and treatment have a positive effect on disease prognosis.
